# Supplementary material for: Screening Mammography and Breast Cancer: Variation in Risk with Rare Deleterious or Predicted Deleterious Variants in DNA Repair Genes
Source: Cancers (Basel). 2025 Mar 21;17(7):1062. doi: 10.3390/cancers17071062 (PMC11987804; doi:10.3390/cancers17071062)
Supplement: Supplementary file 1 [file cancers-17-01062-s001.zip › cancers-3485388-supplementary.pdf]

Eligibility criteria for admission of breast cancer patients to family cancer clinics

The elements that would result in a family cancer clinic consultation are the presence of several cases of breast cancer in a family branch, whether on the paternal or maternal side; early onset of cancer; multifocal and / or bilateral cancer; occurrence of ovarian cancer; occurrence of breast cancer (BC) in men; more rarely, occurrence of pancreatic cancer, melanoma or prostate cancer in the family.

Study population and design

Index cases (and their affected sisters) were identified through the French family cancer clinics of the Groupe Génétique et Cancer (Unicancer) (i.e. 42 centers) and were eligible when diagnosed with infiltrating mammary or ductal adenocarcinoma, were negative for *BRCA1* and *BRCA2* mutations, and had a sister with BC. The mutation screening strategy was similar for all the clinics. Two types of controls were included: unrelated controls and unaffected sisters. The unrelated controls were selected from among the unaffected friends and/or colleagues of the cases. The year of birth of controls was matched to that of the case (±3 years). The parents, brothers and unaffected sisters of the index case were also contacted, when possible. Geneticists at family cancer clinics identified index cases and invited them to participate in GENESIS by referring them to the coordinating center at Institut Curie (Paris, France). The coordinating center organized the inclusion of index cases, family members and their unrelated controls; the collection of blood samples from all participants by sending them a prescription for blood sampling, a letter for the medical analysis laboratory or the nurse who took the blood sample, and appropriate prepaid packaging for shipment of the samples directly to the biological resource center at the Centre Léon Bérard (Lyon, France).

All women completed a questionnaire on environmental, lifestyle and reproductive factors, and family history of cancer. Only women reporting European ancestry (i.e. over 95% of the study population) were considered for this study.

Covariables for logistic regression analyses adjustments.

Analyses were adjusted for age at censoring, which was calculated as the age at diagnosis for cases, and the age at interview for controls. Other adjustment variables were education level (not graduated, basic level, intermediate/high level), birth cohort (≤1945; 1946-1959; ≥1960), body mass index at diagnosis for cases, and at interview for controls (<18.5; 18.5-24.99; 25-29.99; ≥30), number of full-term pregnancies (nulliparous; 1-2; >2), smoking (no; current; past), chest X-ray exposures at least one year before censoring (ever/never), being eligible for the national screening i.e. being born after 1930 and 50 years and older (yes/no) and number of relatives with BC at censure (0;1;≥ 2).

We also considered procedures where the thoracic region was exposed such as conventional radiography, fluoroscopy, computed tomography, and scintigraphy at least one year before censoring (ever/never) as adjustment covariates.
We also adjusted for the number of mammograms (<10 vs. ≥10) when relevant.
Additionally, we adjusted for carrying at least one rare variant in the DNA repair gene groups other than the analyzed gene group.

For the family history of BC variable, we considered the number of reported first- or second- degree relatives affected with BC. Since index cases had an affected sister by design, we excluded one affected sister from the family history count to assess BC family history cases when all affected sisters were diagnosed with BC before the date of the index case's BC diagnosis. Then, we classified BC family history as no affected relatives, at least one affected relative, or two or more affected relatives.

Missing was included in reference categories for each variable of adjustment when no multiple imputation analyses.

Multiple imputation
To evaluate the effect of missing information on the observed results, we performed multiple imputations using the chained equations method (MICE) [27,28] as implemented in STATA [18]. This method uses a Gibbs-like algorithm [29] to obtain 100 imputed datasets with complete observations for each outcome. ORs estimated on the imputed data sets were pooled together using Rubin's rules to obtain valid statistical inferences [30].

Sensitivity analyses
As the DNA repair gene groups were defined using *a priori* bounds for ORs, we performed sensitivity analyses using different bounds, i.e. Group 'Reduced': OR<0.8 or <1.0; Group 'Independent': 0.8≤OR≤1.2 or OR=1.0; Group 'Increased': OR>1.2 or >1.0.

Supplementary tables

Supplemental Table S1. Association of rare coding variants with breast cancer, for the 113 DNA repair genes sequenced in the GENESIS population [15,16].

Gene	Control carriers	Case carriers	Any variant OR[a] (95%CI)	P-value	Group[b]
BABAM1/MERIT40	4	2	0.5 (0.1, 2.8)	0.43	Reduced
BACH1	22	21	0.8 (0.4, 1.5)	0.55	Reduced
BRCC3/BRCC36	3	1	0.3 (0.0, 3.2)	0.34	Reduced
BRE	11	7	0.6 (0.2, 1.6)	0.35	Reduced
CDH1	14	11	0.8 (0.4, 1.7)	0.54	Reduced
CDKN1A	13	11	0.8 (0.4, 1.9)	0.66	Reduced
COBRA1	6	4	0.7 (0.2, 2.4)	0.55	Reduced
DLG1	25	15	0.6 (0.3, 1.2)	0.12	Reduced
ESR1	10	6	0.6 (0.2, 1.5)	0.26	Reduced
EXO1	45	37	0.8 (0.5, 1.3)	0.41	Reduced
FAM175A/ABRAXAS	7	5	0.7 (0.2, 2.3)	0.56	Reduced
FANCA	19	15	0.8 (0.4, 1.6)	0.53	Reduced
FANCD2	20	15	0.7 (0.4, 1.4)	0.33	Reduced
FANCF	6	5	0.8 (0.3, 2.8)	0.77	Reduced
FANCG	6	4	0.7 (0.2, 2.4)	0.55	Reduced
FANCI	22	13	0.6 (0.3, 1.2)	0.13	Reduced
IRS2	13	9	0.7 (0.3, 1.5)	0.33	Reduced
KIAA1967	22	17	0.8 (0.4, 1.5)	0.41	Reduced
LIG4	15	13	0.7 (0.3, 1.6)	0.46	Reduced
MLH3	21	10	0.5 (0.2, 1.0)	0.06	Reduced
MUS81	10	7	0.6 (0.2, 1.6)	0.30	Reduced
MYC	2	2	0.8 (0.1, 5.5)	0.78	Reduced
NAT1	3	1	0.4 (0.0, 3.4)	0.37	Reduced
PMS2	22	16	0.7 (0.3, 1.3)	0.24	Reduced
POLH	13	5	0.4 (0.1, 1.1)	0.07	Reduced
POLQ	43	36	0.8 (0.5, 1.2)	0.43	Reduced
PRKAA2	8	3	0.4 (0.1, 1.5)	0.16	Reduced
RAD51D/RAD51L3	9	4	0.4 (0.1, 1.4)	0.17	Reduced
RAD54L	21	12	0.6 (0.3, 1.2)	0.14	Reduced
RTEL1	20	8	0.4 (0.2, 0.9)	0.03	Reduced
TIMELESS	28	23	0.8 (0.5, 1.4)	0.44	Reduced
TP53BP1	27	21	0.8 (0.5, 1.5)	0.51	Reduced
TP63	6	3	0.5 (0.1, 2.0)	0.33	Reduced
TTI2	9	7	0.8 (0.3, 2.2)	0.67	Reduced
WDR48	15	10	0.7 (0.3, 1.5)	0.36	Reduced
XRCC1	31	21	0.7 (0.4, 1.2)	0.14	Reduced
APEX1	10	10	1.0 (0.4, 2.4)	0.98	Independent
AR	32	31	1.0 (0.6, 1.6)	0.94	Independent
ATR	30	29	1.0 (0.6, 1.6)	0.92	Independent
BAP1	4	4	1.0 (0.3, 4.0)	1.00	Independent

Gene	Control carriers	Case carriers	Any variant OR[a] (95%CI)	P-value	Group[b]
BLM	35	31	0.9 (0.5, 1.4)	0.59	Independent
CDC27	12	12	1.0 (0.5, 2.3)	0.98	Independent
CDKN2A	3	3	1.0 (0.2, 4.9)	0.99	Independent
EIF4G1	26	29	1.1 (0.7, 1.9)	0.67	Independent
EP300	21	18	0.9 (0.5, 1.6)	0.66	Independent
ERCC6	47	53	1.1 (0.8, 1.7)	0.55	Independent
FANCB	9	9	0.9 (0.4, 2.4)	0.87	Independent
FANCC	11	10	0.9 (0.4, 2.2)	0.87	Independent
FANCE	11	10	0.9 (0.4, 2.2)	0.86	Independent
FANCL	9	10	1.1 (0.4, 2.8)	0.84	Independent
FLNA	25	24	1.0 (0.6, 1.7)	0.94	Independent
MAGI3	26	28	1.1 (0.6, 1.8)	0.82	Independent
MAST2	46	50	1.1 (0.7, 1.7)	0.66	Independent
MCM4	25	29	1.1 (0.7, 1.9)	0.70	Independent
MCPH1	27	28	1.0 (0.6, 1.8)	0.92	Independent
MDC1	24	22	0.9 (0.5, 1.7)	0.81	Independent
MSH2	18	17	0.9 (0.5, 1.8)	0.76	Independent
MSH6	16	16	0.9 (0.5, 1.9)	0.86	Independent
NBN	26	27	1.0 (0.6, 1.8)	0.87	Independent
PHLPP2	32	34	1.0 (0.6, 1.7)	0.97	Independent
POLK	22	22	1.0 (0.5, 1.8)	0.99	Independent
RAD51B/REC2/RAD51L1	6	5	0.9 (0.3, 2.8)	0.80	Independent
RECQL4	49	55	1.1 (0.8, 1.7)	0.59	Independent
RINT1	8	8	1.0 (0.4, 2.8)	0.95	Independent
SETX	25	24	1.0 (0.6, 1.7)	0.91	Independent
TELO2	17	18	1.1 (0.5, 2.1)	0.89	Independent
XRCC2	7	6	0.9 (0.3, 2.7)	0.84	Independent
APLF	7	11	1.5 (0.6, 3.9)	0.40	Increased
ATM	40	77	1.9 (1.3, 2.9)	0.001	Increased
BARD1	7	9	1.3 (0.5, 3.6)	0.59	Increased
BRIP1/FANCJ	16	25	1.5 (0.8, 2.8)	0.25	Increased
CHEK1	4	6	1.2 (0.3, 4.5)	0.75	Increased
CHEK2	22	62	3.0 (1.9, 5.0)	0.00001	Increased
CHGB	9	11	1.2 (0.5, 3.0)	0.65	Increased
DCLRE1C	9	14	1.6 (0.7, 3.7)	0.28	Increased
DGKZ	33	38	1.2 (0.7, 1.9)	0.52	Increased
ERCC2	17	27	1.6 (0.9, 3.0)	0.13	Increased
EYA3	6	7	1.2 (0.4, 3.5)	0.77	Increased
FANCM	23	38	1.7 (1.0, 2.8)	0.06	Increased
FEN1	6	7	1.2 (0.4, 3.6)	0.74	Increased
FOXO1	6	7	1.8 (0.5, 6.0)	0.38	Increased
FOXO3	0	8	7.0 [c]	-	Increased
FOXO4	0	4	3.5 [c]	-	Increased
MAST1	8	17	2.2 (0.9, 5.1)	0.07	Increased
MCM7	10	18	1.8 (0.8, 4.0)	0.13	Increased
MLH1	15	19	1.3 (0.6, 2.5)	0.52	Increased

Gene	Control carriers	Case carriers	Any variant OR[a] (95%CI)	P-value	Group[b]
MRE11A	12	14	1.2 (0.6, 2.6)	0.64	Increased
MSH3	25	30	1.2 (0.7, 2.1)	0.49	Increased
NTHL1	18	22	1.2 (0.6, 2.2)	0.65	Increased
NUMA1	36	51	1.4 (0.9, 2.2)	0.12	Increased
PALB2	9	30	3.5 (1.7, 7.5)	0.001	Increased
PIK3R1	1	4	4.3 (0.5, 38.3)	0.20	Increased
PMS1	6	10	1.5 (0.6, 4.3)	0.41	Increased
PPM1D	4	6	1.5 (0.4, 5.4)	0.53	Increased
PTEN	0	4	4.3[c]	-	Increased
RAD50	30	37	1.2 (0.7, 2.0)	0.44	Increased
RAD51C	7	10	1.5 (0.6, 4.0)	0.41	Increased
RAD9B	4	6	1.5 (0.4, 5.2)	0.55	Increased
RECQL5	20	29	1.5 (0.9, 2.7)	0.14	Increased
REV3L	31	39	1.3 (0.8, 2.1)	0.30	Increased
RNF168	13	16	1.2 (0.6, 2.6)	0.59	Increased
RPA1	9	14	1.5 (0.7, 3.6)	0.32	Increased
SLX4/FANCP	36	44	1.2 (0.8, 1.9)	0.38	Increased
STK11	1	2	2.1 (0.2, 22.9)	0.55	Increased
TGFB1	5	9	1.6 (0.5, 4.9)	0.38	Increased
TOP3A	22	31	1.4 (0.8, 2.5)	0.23	Increased
TP53	3	6	2.0 (0.5, 8.0)	0.34	Increased
TSC2	45	56	1.3 (0.9, 1.9)	0.23	Increased
TTI1	26	30	1.2 (0.7, 2.0)	0.57	Increased
UIMC1/RAP80	12	15	1.2 (0.6, 2.7)	0.58	Increased
USP8	9	16	1.7 (0.7, 3.8)	0.23	Increased
WRN	47	59	1.3 (0.9, 1.9)	0.23	Increased
XRCC3	4	7	1.8 (0.5, 6.2)	0.36	Increased

Abbreviations: OR (95% CI): odds ratio (95% confidence interval)

[a] Reference group: non-carrier of a variant in the tested gene.

[b] Group 'Reduced': OR <0.9; Group 'Independent': 0.9≤ OR ≤1.1; Group 'Increased': OR >1.1.

[c] 1 was added in the empty box to compute the OR.

Supplemental Table S2. Sensitivity analyses with varying bounds of ORs for the definition of genetic variant group: effect of lifetime mammography exposure (any exposure) on breast cancer risk according to the number of exposures, the age at first exposure and time since first exposure

	Group "Reduced"				Group "Independent"				Group "Increased"			
	Number of				Number of				Number of			
	Cases	Controls	OR[a]	95%CI	Cases	Controls	OR[a]	95%CI	Cases	Controls	OR[a]	95%CI
	OR<0.8				0.8<=OR<=1.2				OR>1.2			
	N = 135	N = 274			N = 608	N = 726			N = 473	N = 357		
**Mammography exposure**												
Never	9	20	1		92	48	1		47	17	1	
Ever	123	251	4.13	1.20-14.2	542	675	1.21	0.69-2.15	424	338	0.78	0.36-1.71
**Number of mammograms**												
0	9	20	1		62	48	1		47	17	1	
1-4	50	96	4.10	1.19-14.2	230	228	1.36	0.76-2.43	175	113	0.86	0.39-1.89
5-9	51	110	4.64	1.14-19.0	182	314	0.85	0.45-1.63	151	157	0.56	1.24-1.33
≥10	22	45	4.99	1.03-24.2	130	133	1.86	0.90-3.82	98	68	1.09	0.42-2.81
**Age at first exposure, years**[c]												
No exposure	9	20	1		62	48	1		47	17	1	
≥50	9	69	1.08	0.21-5.65	68	149	1.14	0.53-2.47	50	88	0.58	0.21-1.56
40-49	53	121	2.61	0.69-9.91	213	335	0.97	0.52-1.82	163	154	0.67	0.29-1.55
30-39	45	47	4.20	1.16-15.1	200	152	1.31	0.72-2.39	163	75	0.81	0.36-1.82
<30	13	11	6.44	1.39-29.7	56	33	1.85	0.86-4.00	43	15	0.96	0.35-2.65
	OR<1				OR=1				OR>1			
	N = 362	N = 525			N = 212	N = 246			N = 656	N = 602		
**Mammography exposure**												
Never	31	39	1		17	16	1		71	36	1	
Ever	327	483	1.91	0.94-3.87	194	229	1.67	0.62-4.48	581	563	1.04	0.59-1.86
**Number of mammograms**												
0	31	39	1		17	16	1		71	36	1	
1-4	142	175	2.05	1.01-4.17	82	77	1.78	0.65-4.87	233	196	1.12	0.62-2.01
5-9	114	215	1.44	0.65-3.16	64	110	1.70	0.57-5.11	203	256	0.85	0.45-1.64
≥10	71	93	2.29	0.96-5.47	48	42	4.14	1.18-14.5	145	111	1.96	0.95-4.02

	Group "Reduced"				Group "Independent"				Group "Increased"			
	Number of				Number of				Number of			
	Cases	Controls	OR[a]	95%CI	Cases	Controls	OR[a]	95%CI	Cases	Controls	OR[a]	95%CI
**Age at first exposure, years**[c]												
No exposure	31	39	1		17	16	1		71	36	1	
≥50	36	118	1.66	0.64-4.30	20	59	1.10	0.30-4.04	68	132	0.81	0.37-1.75
40-49	133	230	1.57	0.73-3.38	80	112	1.19	0.41-3.45	226	275	0.85	0.45-1.59
30-39	116	102	2.03	0.96-4.27	71	45	1.95	0.68-5.56	222	125	1.13	0.61-2.07
<30	38	23	2.41	0.96-6.01	23	10	3.65	0.99-13.4	58	24	1.38	0.63-3.01

Abbreviations: OR (95% CI): odds ratio (95% confidence interval); missing values coded as additional category.

[a] Adjusted for age at censoring, birth cohort (≤1945; 1946-1959; ≥1960), number of full-term pregnancies (>2; 1-2; 0), educational level (intermediate/Increased; basic; no graduated), BMI (18.5-24.9; <18.5; ≥25), smoking (no; current; past), Chest X-ray exposure (ever vs. never), being eligible for the national screening (yes/no), number of relatives with breast cancer (0; 1; ≥ 2) and the two other DNA repair genes groups, with missing included in reference categories for each variable.

Chest X-ray exposure includes pulmonary radiological examinations in the field of preventive / occupational medicine or for lung disease, preoperative radiological examinations, and radiological examinations of heart and thoracic vessels for all the reported procedures.

[c] Adjusted as [a] plus number of mammography (<10; ≥10)

Supplemental Table S3. Sensitivity analyses with varying bounds of ORs for the definition of genetic variant group: effect of lifetime mammography exposure (any exposure) on breast cancer risk according to the number of exposures, the age at first exposure and time since first exposure (Multiple Imputation)

	Group "Reduced"				Group "Independent"				Group "Increased"			
	Number of				Number of				Number of			
	Cases	Controls	OR[a]	95%CI	Cases	Controls	OR[a]	95%CI	Cases	Controls	OR[a]	95%CI
	OR<0.8				0.8<=OR<=1.2				OR>1.2			
	N = 135	N = 274			N = 608	N = 726			N = 473	N = 357		
**Mammography exposure**												
Never	9	20	1		92	48	1		47	17	1	
Ever	123	251	3.93	1.15-13.4	542	675	1.18	0.67-2.10	424	338	0.77	0.35-1.70
**Number of mammograms**												
0	9	20	1		62	48	1		47	17	1	
1-4	50	96	3.87	1.13-13.3	230	228	1.32	0.74-2.36	175	113	0.84	0.38-1.87
5-9	51	110	4.34	1.07-17.6	182	314	0.84	0.44-1.61	151	157	0.58	0.24-1.39
≥10	22	45	4.63	0.96-22.3	130	133	1.82	0.89-3.75	98	68	1.15	1.01-1.09
**Age at first exposure, years**[c]												
No exposure	9	20	1		62	48	1		47	17	1	
≥50	9	69	1.14	0.22-5.97	68	149	1.11	0.51-2.42	50	88	0.59	0.22-1.63
40-49	53	121	2.60	0.69-9.80	213	335	0.96	0.51-1.79	163	154	0.68	0.29-1.61
30-39	45	47	4.11	1.15-14.7	200	152	1.26	0.69-2.30	163	75	0.80	0.35-1.80
<30	13	11	6.13	1.34-28.2	56	33	1.70	0.78-3.69	43	15	0.86	0.31-2.40
	OR<1				OR=1				OR>1			
	N = 362	N = 525			N = 212	N = 246			N = 656	N = 602		
**Mammography exposure**												
Never	31	39	1		17	16	1		71	36	1	
Ever	327	483	1.86	0.92-3.77	194	229	1.65	0.61-4.45	581	563	1.01	0.56-1.81
**Number of mammograms**												
0	31	39	1		17	16	1		71	36	1	
1-4	142	175	1.98	0.97-4.05	82	77	1.75	0.64-4.81	233	196	1.07	0.59-1.94
5-9	114	215	1.41	0.64-3.11	64	110	1.69	0.56-5.09	203	256	0.84	0.44-1.63
≥10	71	93	2.24	0.94-5.36	48	42	4.06	1.15-14.3	145	111	1.94	0.94-4.00

	Group "Reduced"				Group "Independent"				Group "Increased"			
	Number of				Number of				Number of			
	Cases	Controls	OR[a]	95%CI	Cases	Controls	OR[a]	95%CI	Cases	Controls	OR[a]	95%CI
**Age at first exposure, years[c]**												
No exposure	31	39	1		17	16	1		71	36	1	
≥50	36	118	1.65	0.63-4.28	20	59	1.01	0.27-3.75	68	132	0.80	0.37-1.74
40-49	133	230	1.55	0.72-3.35	80	112	1.16	0.40-3.36	226	275	0.84	0.44-1.58
30-39	116	102	1.94	0.92-4.08	71	45	1.88	0.65-5.38	222	125	1.08	0.59-1.99
<30	38	23	2.23	0.89-5.58	23	10	3.20	0.86-11.8	58	24	1.25	0.57-2.75

Abbreviations: OR (95% CI): odds ratio (95% confidence interval).

[a] Adjusted for age at censoring, birth cohort (≤1945; 1946-1959; ≥1960), number of full-term pregnancies (>2; 1-2; 0), educational level (intermediate/Increased; basic; no graduated), BMI (18.5-24.9; <18.5; ≥25), smoking (no; current; past), Chest X-ray exposure (ever vs. never), being eligible for the national screening (yes/no), number of relatives with breast cancer (0; 1; ≥ 2) and two other DNA repair genes groups. Chest X-ray exposure includes pulmonary radiological examinations in the field of preventive / occupational medicine or for lung disease, preoperative radiological examinations, and radiological examinations of heart and thoracic vessels for all the reported procedures. [c] Adjusted as [a] plus number of mammography (<10;≥10)

Supplemental Table S4. Number of breast cancers in the family at censure according to age at mammography first exposure

	Number of breast cancers in the family at censure															
	**None**				**1**				**≥ 2**				**Total**			
	Cases		Controls		Cases		Controls		Cases		Controls		Cases		Controls	
	N	%	N	%	N	%	N	%	N	%	N	%	N	%	N	%
**Age at mammography first exposure (years)**																
≥ 50	53	11.8	237	25.6	65	12.1	50	19.3	29	11.0	8	13.8	147	11.8	295	23.8
40-49	193	43.1	463	50.1	196	36.5	130	50.2	98	37.1	25	43.1	487	39.1	618	49.8
30-39	162	36.2	184	19.9	215	40.0	63	24.3	106	40.2	22	37.9	483	38.7	269	21.7
< 30	40	8.9	40	4.3	61	11.4	16	6.2	31	11.7	3	5.2	132	10.5	59	4.7
**Total**	448		924		537		259		264		58		1249		1241	

Chi^2=23.3 (23 df); p=0.44

Supplemental Table S5. Characteristics of GENESIS participants according to DNA repair gene groups

	Group "Reduced"				Group "Independent"				Group "Increased"			
	Cases		Controls		Cases		Controls		Cases		Controls	
	N	%	N	%	N	%	N	%	N	%	N	%
**Birth cohort**												
≤1945	57	25.1	85	24.9	68	22.0	87	22.4	86	22.6	79	22.1
1946-59	121	53.3	173	50.7	173	56.0	208	53.5	207	54.3	195	54.6
≥1960	49	21.6	83	24.3	68	22.0	94	24.2	88	23.1	83	23.3
**Age at censoring, years**												
Mean (SD)	51.75 (8.82)		56.76 (9.36)		51.54 (9.31)		56.33 (9.27)		51.61 (9.07)		56.47 (9.23)	
**Education level**												
Intermediate/Increased	117	51.5	222	65.1	171	55.3	260	66.8	191	50.1	236	66.1
Basic	102	44.9	114	33.4	126	40.8	125	32.1	174	45.7	116	32.5
No graduated	8	3.5	5	1.5	12	3.9	4	1.0	16	4.2	5	1.4
**Being eligible for the national screening**												
No	92	40.5	89	26.1	141	45.6	94	24.2	170	44.6	85	23.8
Yes	135	59.5	252	73.9	168	54.4	295	75.8	211	55.4	272	76.2
**Number of breast cancers in the family at censure**												
None	66	29.1	268	78.6	99	32.0	286	73.5	138	36.2	266	74.5
1	110	48.5	63	18.5	152	49.2	76	19.5	163	42.8	73	20.5
≥2	51	22.5	10	2.9	58	18.8	27	7.0	80	21.0	18	5.0

Supplemental Table S6. Cross table between lifetime number of mammograms and lifetime number of views.

	Lifetime number of views							
	Cases				Controls			
	Range[a]	Mean	SD	Missing[b] %	Range	Mean	SD	Missing[c] %
**Lifetime number of mammograms**								
1-4	1-36	11.1	6.20	198 (32.2%)	1-27	11.6	5.76	90 (18.6%)
5-9	5-72	30.8	11.1	113 (27.6%)	9-51	30.0	9.51	74 (13.8%)
≥10	4-217	67.8	30.6	81 (32.7%)	5-166	56.9	21.4	53 (22.8%)

[a] lifetime number of views was calculated by summing the number of views filled up in the questionnaire over all the mammograms even when numbers of views were missing for some mammograms. This explains inconsistencies observed between the lower bound of the number of views ranges and the number of mammograms.
[b] number of cases with no data on the numbers of views
[c] number of controls with no data on the numbers of views

Note: To better assess the effect of radiation from mammograms, considering the number of views might be more accurate; however, we chose not using this information because of an elevated rate of participants who did not fill up the number of views for any of the mammograms (up to 25% among cases). This rate of missing data was even more elevated when the rate of mammograms without information on the number of views was considered (about a third among cases) leading to increased rate of misclassification.
